# Interaction of Optimal Cerebral Perfusion Pressure with Early Brain Injury and its Impact on Ischemic Complications and Outcome Following Aneurysmal Subarachnoid Hemorrhage

**DOI:** 10.1007/s12028-023-01822-1

**Published:** 2023-09-19

**Authors:** Vesna Malinova, Beate Kranawetter, Sheri Tuzi, Onnen Moerer, Veit Rohde, Dorothee Mielke

**Affiliations:** 1 Department of Neurosurgery, University Medical Center Göttingen, Georg-August-University, Robert-Koch-Straße 40, 37075 Göttingen, Germany; 2 Department of Anesthesiology, University Medical Center Göttingen, Göttingen, Germany

**Keywords:** Early brain injury, Optimal cerebral perfusion pressure, Subarachnoid hemorrhage

## Abstract

**Background:**

Cerebral autoregulation is impaired early on after aneurysmal subarachnoid hemorrhage (aSAH). The study objective was to explore the pressure reactivity index (PRx) and cerebral perfusion pressure (CPP) in the earliest phase after aneurysm rupture and to address the question of whether an optimal CPP (CPPopt)–targeted management is associated with less severe early brain injury (EBI).

**Methods:**

Patients with aSAH admitted between 2012 and 2020 were retrospectively included in this observational cohort study. The PRx was calculated as a correlation coefficient between intracranial pressure and mean arterial pressure. By plotting the PRx versus CPP, CPP correlating the lowest PRx value was identified as CPPopt. EBI was assessed by applying the Subarachnoid Hemorrhage Early Brain Edema Score (SEBES) on day 3 after ictus. An SEBES ≥ 3 was considered severe EBI.

**Results:**

In 90 of 324 consecutive patients with aSAH, intracranial pressure monitoring was performed ≥ 7 days, allowing for PRx calculation and CPPopt determination. Severe EBI was associated with larger mean deviation of CPP from CPPopt 72 h after ictus (*r* = 0.22, *p* = 0.03). Progressive edema requiring decompressive hemicraniectomy was associated with larger deviation of CPP from CPPopt on day 2 (*r* = 0.23, *p* = 0.02). The higher the difference of CPP from CPPopt on day 3 the higher the mortality rate (*r* = 0.31, *p* = 0.04).

**Conclusions:**

Patients with CPP near to the calculated CPPopt in the early phase after aSAH experienced less severe EBI, less frequently received decompressive hemicraniectomy, and exhibited a lower mortality rate. A prospective evaluation of CPPopt-guided management starting in the first days after ictus is needed to confirm the clinical validity of this concept.

## Introduction

Aneurysmal subarachnoid hemorrhage (aSAH) induces a sudden increase in intracranial pressure (ICP), whose extent varies depending on the amount of extravasated blood and the individual intracranial compliance. This leads to a depletion of cerebral perfusion pressure (CPP), which in turn promotes cell death and causes poor outcome. Subarachnoid hemorrhage–induced brain damage has a complex pathophysiology, whereas previous aSAH research essentially differentiated between early and delayed brain injury. Experimental studies suggest that CPP depletion at the time of aneurysm rupture potentially triggers processes that eventually result in early brain injury (EBI) after aSAH [1]. The EBI concept has been initially extrapolated from experimental data and is still insufficiently explored in clinical practice. Although aSAH research has been mainly focused on macrovasospasm for a long time, insights from recent studies have acknowledged the role of cerebral microcirculation in this context, a primary determinant of cerebrovascular resistance and a key element of automated cerebral perfusion control, i.e., cerebral autoregulation (CA) [2]. Patients with aSAH may have a reduced cerebrovascular reserve capacity and are subsequently more likely vulnerable to hypotension than healthy individuals [2]. Using the pressure reactivity index (PRx), optimal CPP (CPPopt) can be calculated, corresponding to CPP within a given range, with the lowest PRx representing an optimal function of CA for the patient. Patients with traumatic brain injury with CPP close to their CPPopt experienced better outcomes than those with large deviations of CPP from CPPopt [3]. The study objective was to explore the PRx and CPP in the earliest phase of aSAH and to address the question of whether a CPPopt-targeted management is associated with less severe EBI and a better outcome. The hypothesis was that negative PRx values reflecting a preserved CA and CPP near the individually calculated CPPopt correlate with less severe EBI and a better functional outcome. On the contrary, positive PRx values and higher deviations of CPP from the ascertained CPPopt have been expected to be found more often in patients experiencing more severe EBI.

## Methods

### Study Population

This is a retrospective observational cohort study of consecutive patients with aSAH treated at our center between January 2012 and December 2020. Inclusion criteria were age ≥ 18 years and confirmed aSAH within 24 h of symptom onset. The diagnosis of aSAH was confirmed by computed tomography (CT) and CT angiography and/or digital subtraction angiography. Patients with other subarachnoid hemorrhage causes were excluded. Another inclusion criterion was a continuous invasive monitoring of ICP and arterial blood pressure for at least 72 h after ictus. The metric data, such as blood pressure and ICP and CPP values, were retrospectively extracted from the IntelliSpace Critical Care and Anesthesia intensive care unit software and were used for the calculation of the PRx and CPPopt. All patients diagnosed with aSAH were treated according to a predefined management protocol at our department. Patients with aSAH were under observation at the intensive care unit for at least 14 days. In patients with ICP monitoring, CPP was continuously monitored, and CPP > 70 mm Hg was maintained according to current guidelines. Mean arterial pressure (MAP) was monitored in all patients via arterial line, and MAP > 65 mm Hg was maintained according to current guidelines. Transcranial Doppler sonography and neurological assessment were performed at least twice a day. In case of clinical deterioration with suspected delayed cerebral ischemia (DCI) or in case of increased blood flow velocities, as measured by transcranial Doppler in the middle cerebral artery, computed tomography perfusion (CTP) including a native CT scan and CT angiography was indicated. In comatose and/or sedated patients, routine CTP was performed on day 3 and day 7 after ictus. If DCI was confirmed, induced hypertension (systolic arterial blood pressure 160–180 mm Hg) was started. In case of severe cerebral vasospasm with corresponding perfusion deficits, conventional angiography was additionally done with chemical or mechanical spasmolysis. Endovascular rescue therapy (i.e., chemical or mechanical spasmolysis) was also indicated in case of DCI refractory to induced hypertension. All patients were treated according to guideline recommendations. The study was approved by the local institutional review board (16/9/20). Because of the retrospective study design, informed consent was not required.

### Calculation of PRx and CPPopt

For multimodal data acquisition, IntelliSpace Critical Care and Anesthesia intensive care unit software was used. For ICP monitoring, an intraparenchymal ICP probe (Codman Microsensors ICP Transducer; Codman & Shurtleff Inc) was inserted into the right frontal lobe. The PRx was continuously monitored and retrospectively calculated as a correlation coefficient between ICP and MAP in a 4-h time window. The PRx ranges from − 1 to 1, whereby PRx values < 0 suggest a preserved CA and PRx values > 0 indicate impaired CA. By plotting the PRx versus CPP, CPP correlating to the lowest PRx value was identified as the CPPopt. Deviations of CPP from CPPopt were calculated, and cases with CPP < CPPopt were documented.

### Primary End Points

EBI was evaluated by applying the Subarachnoid Hemorrhage Early Brain Edema Score (SEBES) [4] on day 3 after ictus. An SEBES ≥ 3 was considered severe EBI. The SEBES was introduced by Ahn et al. [4] as a score for the assessment of EBI based on radiological criteria. The score was internally validated by the authors and applied in other publications. An SEBES score of 3 or 4 was an independent predictor of DCI and unfavorable outcome. The SEBES was assessed by only one person (ST) to avoid discrepancy during interpretation of the individual items. Progressive brain edema with refractory ICP and a consecutive indication for decompressive hemicraniectomy (DHC) were regarded as surrogate parameters for the most severe EBI manifestation. Functional outcome was assessed using the modified Rankin scale (mRS) at 3 months follow-up. An mRS score ≤ 2 was regarded as favorable outcome. The mRS was assessed during the follow-up examination of the patients that was scheduled in the outpatient clinic 3 months after onset. The mRS assessment was done by only one person (VM) to avoid a bias. DCI was defined as neurological deterioration (after excluding other possible causes, such as hydrocephalus, infection, epileptic seizure) and/or increased blood flow velocities in the middle cerebral artery, measured by transcranial Doppler sonography and angiographic vasospasm, depicted by CT angiography or digital subtraction angiography associated with corresponding perfusion deficits on CTP. Because this was a retrospective analysis, the DCI diagnosis was based on reviews of medical records, which were checked by two independent investigators (BK and VM). Delayed infarction due to DCI was defined as newly detected infarction on the follow-up CT scan after excluding treatment-related infarctions by performing a CT scan 24 h after aneurysm occlusion.

### Statistical Analysis

Statistical analyses were performed by means of the GraphPad Prism software (version 9, GraphPad Software, San Diego, CA). For the presentation of baseline data, descriptive statistics and frequency distribution analysis was done. Continuous variables are depicted as mean ± standard deviation, and categorical variables are depicted as frequency or percentages. Descriptive statistics was used for calculation of baseline characteristics in the study population. Correlation analysis was performed to assess the correlation of the deviation of CPP from CPPopt with the need for DHC due to progressive edema with refractory ICP, with the occurrence of delayed infarctions, and with functional outcome at 3 months’ follow-up. The parameters that were significant in the univariate analysis were included in a multivariate logistic regression analysis, in which parameters were independent predictors.

## Results

### Study Population

**Fig. 1:**
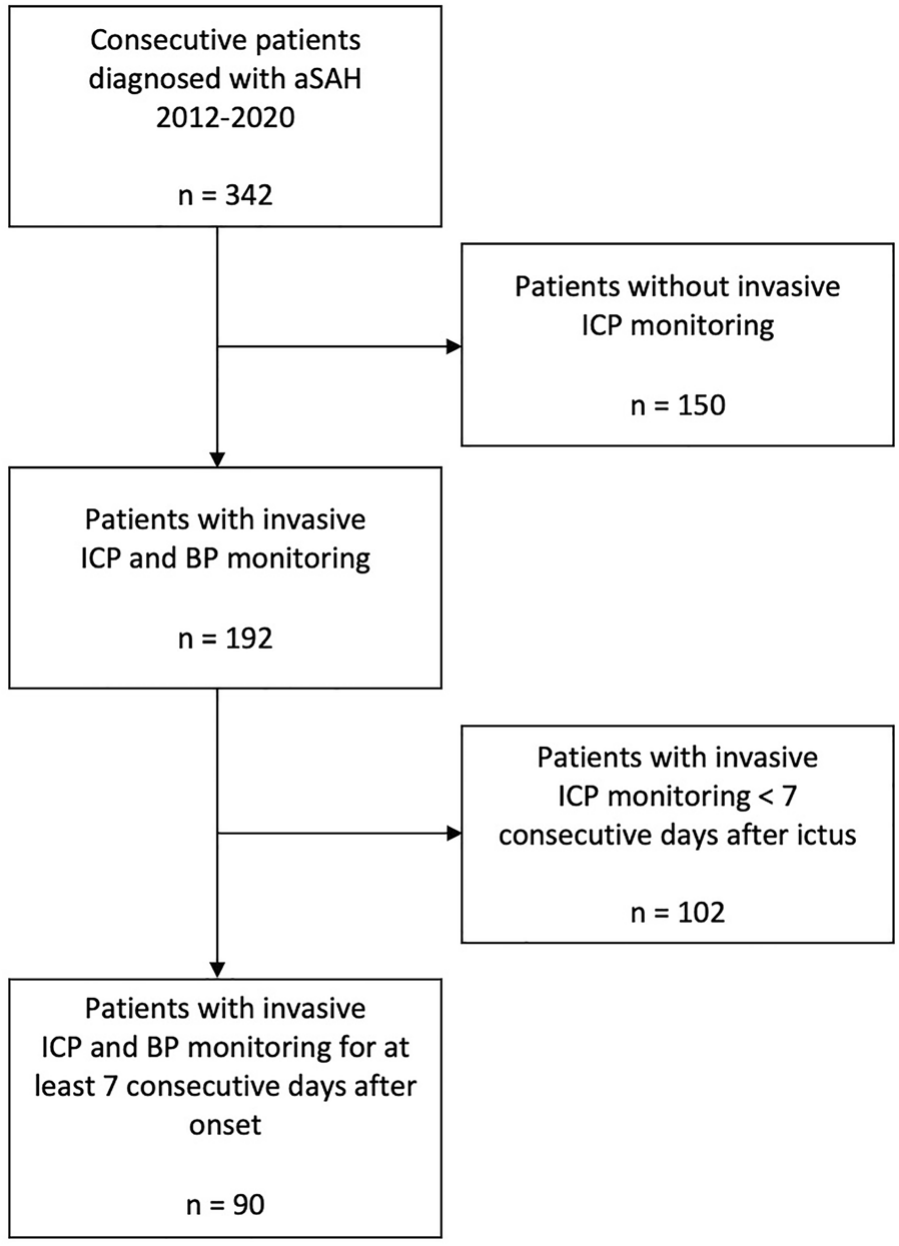
Flow diagram showing the constitution of the study population after applying the inclusion and exclusion criteria. aSAH aneurysmal subarachnoid hemorrhage, BP blood pressure, ICP intracranial pressure

In 90 of 324 consecutive patients with aSAH (28%), ICP monitoring was performed for ≥ 7 days after ictus, allowing for the calculation of the PRx and determination of CPPopt (Fig. 1). The mean age of included patients was 54.2 ± 11.9 years (range 28–80), of whom 64% (58 of 90) were female. Baseline characteristics of the patient population are revealed in Table 1.

**Table 1:** Patients’ characteristics

	Value
Number of analyzed patients	90
Mean age ± SD in years	54.2 ± 11.9
Sex	
Male	36% (32/90)
Female	64% (58/90)
WFNS grade	
I–III	36% (32/90)
IV–V	64% (58/90)
Fisher grade	
1–2	0% (0/90)
3–4	100% (90/90)
Ruptured aneurysm localization	
Anterior circulation	83% (75/90)
Posterior circulation	17% (15/90)
Aneurysm treatment	
Clipping	56% (50/90)
Coiling	44% (40/90)

WFNS, xxx

### EBI vs. PRx and CPPopt Within 72 h after Aneurysm Rupture

**Fig. 2:**
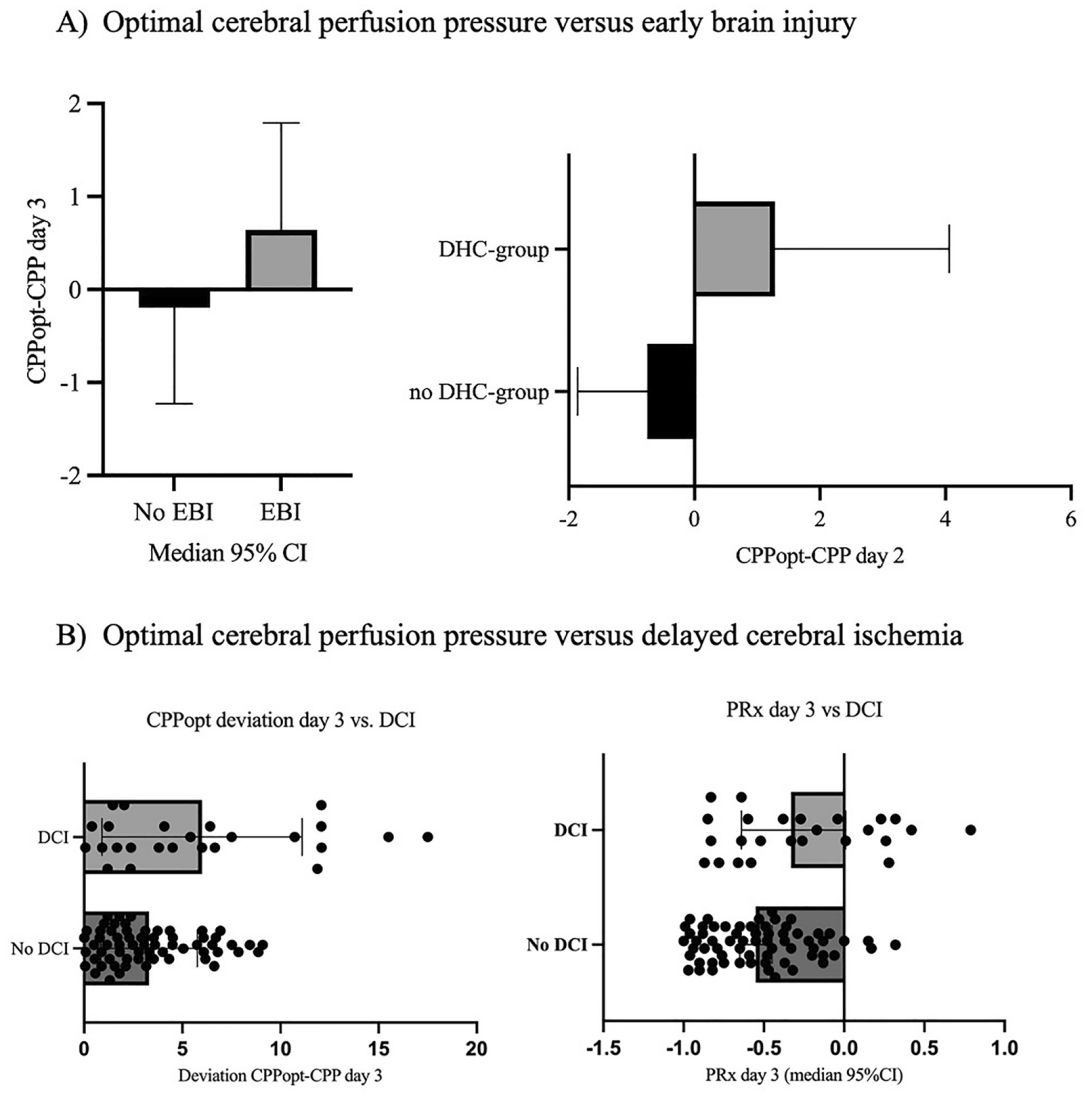
**a** Correlation of optimal cerebral perfusion pressure (CPPopt) with early brain injury (EBI) and decompressive hemicraniectomy (DHC). **b** Correlation of CPPopt and pressure reactivity index (PRx) with delayed cerebral ischemia (DCI). CI confidence interval

**Fig. 3:**
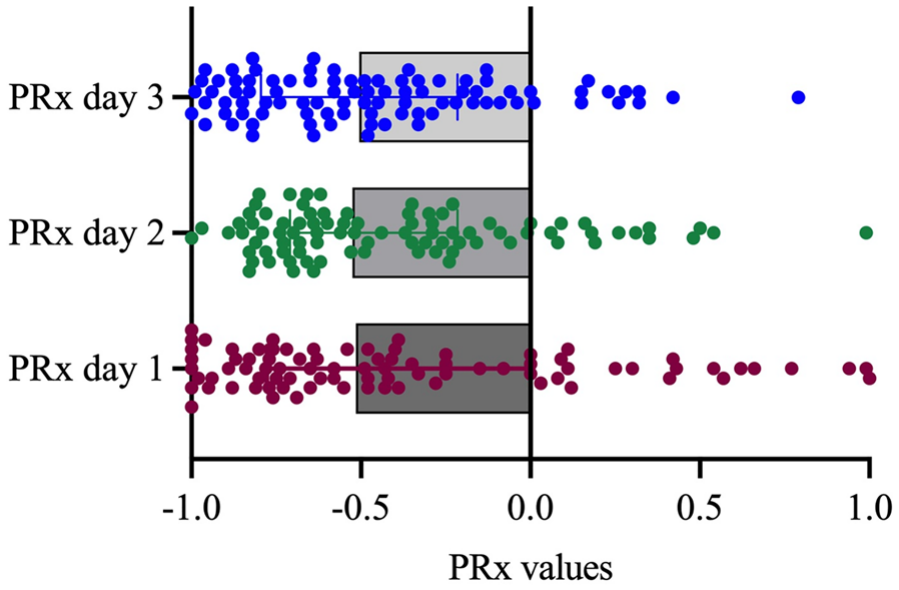
Distribution of pressure reactivity index (PRx) values in the study population on day 1, day 2, and day 3 after ictus

**Fig. 4:**
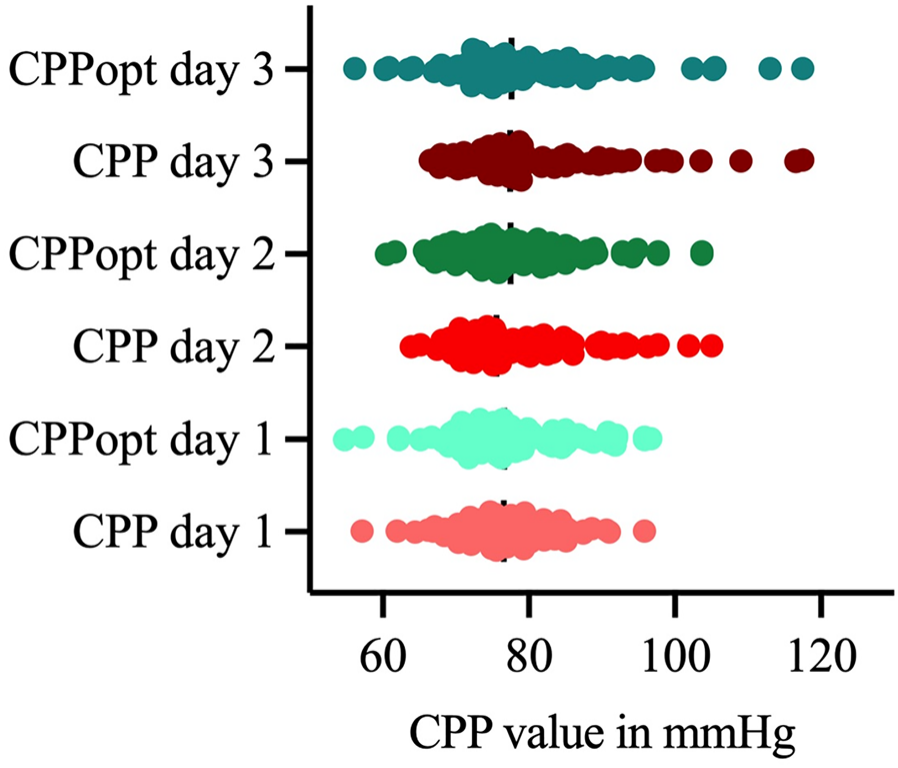
Distribution of cerebral perfusion pressure (CPP) and optimal cerebral perfusion pressure (CPPopt) in the study population on day 1, day 2, and day 3 after ictus

On day 3 of aSAH, an SEBES ≥ 3 was present in 51% (46 of 90) of the patients. An SEBES ≥ 3 was associated with a larger mean deviation of CPP from CPPopt within 72 h after aneurysm rupture (*r* = 0.22, 95% CI 0.01–0.40, *p* = 0.03). DHC was performed in 23% (21 of 90) of the included patients owing to progressive edema with refractory ICP. The median time from ictus to DHC was 4 days (95% CI 3–5). Progressive edema and refractory ICP with a subsequent indication for DHC were associated with larger deviation of CPP from CPPopt on day 2 (*r* = 0.23, 95% CI 0.02–0.42, *p* = 0.02), whereby a CPP < CPPopt on day 2 correlated with the indication for DHC (*r* = 0.22, 95% CI 0.01–0.41, *p* = 0.03), (Fig. 2a). Considering the deviation of CPP from CPPopt during the first 3 days after ictus, there was a significant correlation of mean deviation with severe EBI (*r* = 0.22, *p* = 0.03) and with an earlier duration from ictus to DHC (*r* =  − 0.42, *p* = 0.04). After performing the multivariate logistic regression analysis, only SEBES ≥ 3 remained a significant predictor of progressive edema requiring DHC (Table 2). The distribution of the PRx, CPP, and CPPopt on day 1, day 2, and day 3 after ictus, respectively, is demonstrated in Figs. 3 and 4.

**Table 2:** Multivariate logistic regression analysis

Variable	Odds ratio	95% CI	*p* value
*Prediction of delayed infarction*			
SEBES day 3	1.99	1.23–3.56	0.008*
PRx day 3	11.12	1.73–95.02	0.01*
PRx day 3 − PRx day 1	1.22	0.42–3.88	0.71
CPPopt − CPP day 3	1.22	1.06–1.43	0.007*
CPPopt − CPP day 2	1.17	0.97–1.43	0.09
*Prediction of poor functional outcome, mRS > 3*			
PRx day 2	4.46	0.57–41	0.15
PRx day 3 − PRx day 1	2.41	0.65–11.8	0.22
CPPopt − CPP day 2	0.94	0.77–1.12	0.51
*Progressive edema with refractory ICP requiring DHC*			
SEBES day 3	0.01	0.0001–0.092	0.003*
CPPopt − CPP day 2	1.10	0.97–1.28	0.12
CPPopt − CPP day 3	1.11	0.97–1.28	0.11

*CI* Confidence interval, *CPP* Cerebral perfusion pressure, *CPPopt* Optimal cerebral perfusion pressure, *DHC* Decompressive hemicraniectomy, *ICP* Intracranial pressure, *mRS* Modified Rankin scale, *PRx* Pressure reactivity index, *SEBES* Subarachnoid Hemorrhage Early Brain Edema Score

^*^Xxx

### Delayed Brain Injury Versus PRx and CPPopt Within 72 h After Aneurysm Rupture

Delayed infarctions developed in 28% (25 of 90) of included patients. A PRx > 0 on day 3 was associated with delayed infarctions (*r* = 0.34, 95% CI 0.14–0.51, *p* = 0.001). A higher PRx on day 3 after ictus was associated with higher incidence of infarctions (*r* = 0.30, 95% CI 0.10–0.48, *p* = 0.003). The difference in PRx between day 3 and day 1 (representing impaired CA) correlated with the incidence of infarctions (*r* = 0.22, 95% CI 0.02–0.42, *p* = 0.03). A larger deviation of CPP from CPPopt on day 2 correlated with higher incidence of infarctions (*r* = 0.34, 95% CI 0.05–0.57, *p* = 0.02), as well as on day 3 (*r* = 0.30, 95% CI 0.00–0.55, *p* = 0.04). The higher the deviation of CPP from CPPopt on day 3 (*r* = 0.28, 95% CI 0.08–0.45, *p* = 0.006) the higher was the incidence of infarctions. Infarctions occurred significantly more often in patients with SEBES ≥ 3 (*r* = 0.25, 95% CI 0.05–0.44,* p* = 0.01) as well as in patients with progressive brain edema requiring DHC (*r* = 0.39, 95% CI 0.20–0.55, *p* = 0.0001) (Fig. 2b). After performing the multivariate logistic regression analysis, SEBES ≥ 3, the PRx on day 3, and deviation of CPP from CPPopt on day 3 remained significant predictors of delayed infarction (Table 2).

### Functional Outcome versus PRx and CPPopt Within 72 h After Aneurysm Rupture

A higher difference of CPP from CPPopt on day 3 was associated with a higher mortality rate (*r* = 0.31, 95% CI 0.00–0.57, *p* = 0.04). The higher the difference of CPP from CPPopt on day 3 the higher the chance of achieving an mRS score > 3 at 3 months’ follow-up (*r* = 0.25, 95% CI 0.03–0.45, *p* = 0.02). Higher PRx values on day 2 correlated with higher mortality rates (*r* = 0.23, 95% CI 0.02–0.43, *p* = 0.03). After performing the multivariate logistic regression analysis, none of the parameters that were significant in the univariate analysis remained significant predictions of outcome (Table 2).

## Discussion

In this study, we found a significant correlation of CPPopt within the first days after aneurysm rupture with less severe EBI, less frequent occurrence of progressive edema requiring DHC, lower incidence of delayed infarctions, and better outcome at 3 months’ follow-up. Evaluation of CA after stroke revealed that CA impairment is not an all-or-none phenomenon but rather is graded and variable among patients [5]. Previous research demonstrated an impaired cerebral autoregulatory capacity in the early phase of aSAH, which in turn has been shown to correlate with neurologic complications on an individual patient level [6]. Patients with impaired CA following aSAH are deemed to be at higher risk for DCI and poor outcome [7]. Indeed, a correlation of disturbed CA with cerebral vasospasm, delayed infarctions, and functional outcome at 6 months’ follow-up was previously shown in the context of aSAH [8]. Assessing the CA profiles in these patients would allow for an optimized guidance of treatment strategies [9]. However, CA indices are still insufficiently explored and implemented in clinical practice. The clinical benefit of CA monitoring and CA-guided therapy still needs to be defined. Currently there are no randomized controlled trials confirming the effectiveness of CPPopt-based management in patients with aSAH that are needed for making the next steps toward an optimal individualized patient management during the acute phase after the bleeding. The concept of CA-oriented therapy postulates that CA is an important self-protecting mechanism by which the restoration of CA after brain injury assures continuity and adequacy of cerebral blood flow (CBF) and in turn reduces the risk for ischemic and hyperemic secondary brain damage [10]. Because no methods for continuous monitoring of CBF are currently available for use in clinical practice, different surrogate parameters for CBF, such as CBF velocity, brain tissue oxygen partial pressure, and regional cerebral oxygen saturation, are instead applied, making the comparability of studies even more difficult [11]. Disturbances of CA occur early on after aneurysm rupture. Consequently, a continuous assessment of CA starting within the first days after onset seems to be indicated in patients with aSAH. Hemodynamic augmentation (i.e., induced hypertension) is currently applied during the peak phase of cerebral vasospasm in patients with aSAH. Nevertheless, a randomized controlled trial failed to demonstrate a significant increase in CBF by conducting induced hypertension with a targeted systolic arterial pressure of 180 mm Hg in patients with aSAH [10]. In this study, neither the functionality of CA nor a history of arterial hypertension was considered on an individual level, which could be one possible explanation for why the pursuit of a common blood pressure target has not provided the expected improvement in CBF. The findings of our study confirm an association of disturbed CA and deviations of CPP from CPPopt in the first days after aSAH onset not only with the severity of EBI but also with DCI incidence and poor neurological outcome. This indicates that an improvement of the hemodynamic situation in this phase may open the possibility to positively impact neurological outcome by early initiation of measures to maintain best possible cerebral perfusion on an individual basis. CPP is regarded as a general indicator of brain perfusion. Therefore, CPP monitoring is an integral part of management protocols for the treatment of patients with high-grade aSAH at the intensive care unit. Current guidelines recommend CPP > 70 mm Hg and MAP > 65 mm Hg in patients with aSAH. The CPP concept does not consider the functional status of CA, which is often disturbed after aSAH. The degree of CA disturbance may vary on an individual basis, depending on the severity of the bleeding, the patient’s age, and various other factors. Continuous monitoring of CA indices and setting of CA-based individualized targets, such as an individually calculated CPPopt, introduces a transition from the one-treatment-fits-all paradigm derived from current guidelines toward a personalized medicine approach tailored to the individual patient.

Before the implementation of CPPopt-targeted management of patients with aSAH in clinical practice, several currently unanswered questions need to be addressed: What is the most appropriate method to continuously monitor CA? What is the most reliable measure to assess the severity of EBI? The SEBES was used in our study as a surrogate parameter for the severity of EBI and was calculated on the third day after aSAH onset and currently represents the most valid method for EBI assessment [4]. Nevertheless, other parameters available within the first 24 h may allow for the use of early measures and have the capacity to possibly ameliorate EBI. Several CA indices for continuous monitoring of CA, such as the mean velocity index, have been evaluated as tools for continuous assessment of CA during the acute management of patients with aSAH in clinical practice [13]. The PRx was applied in this study to ascertain CA capacity and is often used for this purpose [14–16]. Hence, the results of this study provide first evidence as a foundation for further prospective evaluation but do not allow for generalization.

The main limitation of the study is the retrospective data analysis. Hence, the CA indices were calculated retrospectively. Another limitation is the relatively small number of patients, as only patients with invasive ICP monitoring for at least 7 days were required for calculation of the PRx and individual determination of CPPopt. However, the patient cohort was extracted from a consecutive aSAH patient cohort treated according to the same protocol. Concerning the functional outcome, no long-term data of later than 3 months after ictus were available for the analysis of this study. Because of the retrospective study design, the findings presented here do not allow for general conclusions regarding causation. Whether deviations of CPP from CPPopt also correlate with long-term outcome needs an evaluation in a future study.

## Conclusions

The findings in our study support the hypothesis that CPPopt-oriented CPP targets starting directly after diagnosis may positively impact the course of disease and outcome after aSAH. Further research with establishment of continuous monitoring and treatment targets would facilitate the implementation of this concept in clinical practice.
